# Respect Is Central: A Critical Review of Implementation Frameworks for Continuous Quality Improvement in Aboriginal and Torres Strait Islander Primary Health Care Services

**DOI:** 10.3389/fpubh.2021.630611

**Published:** 2021-07-16

**Authors:** Michelle Redman-MacLaren, Nalita Nungarrayi Turner (Anmatyerre/Jaru), Judy Taylor, Alison Laycock, Kristina Vine, Quitaysha Thompson (Gurindji), Sarah Larkins, Karen Carlisle, Sandra Thompson, Ross Bailie, Veronica Matthews (Quandamooka)

**Affiliations:** ^1^College of Medicine and Dentistry, James Cook University, Townsville, OLD, Australia; ^2^University Centre for Rural Health, University of Sydney, Lismore, NSW, Australia; ^3^Western Australia Centre for Rural Health, University of Western Australia, Geraldton, WA, Australia

**Keywords:** implementation frameworks, continuous quality improvement, Aboriginal and Torres Strait Islander, primary health care, community health, Indigenous

## Abstract

**Background:** Primary health care (PHC) services are complex systems, shaped by an interplay of factors at individual, organisational and broader system levels. For Aboriginal and Torres Strait Islander PHC services, closer relationships with the people they serve, local knowledge of community, and cultural awareness are critical. Continuous quality improvement (CQI) has proven to be an effective process for identification of priority issues in health care delivery and for instigating the design, implementation and evaluation of improvement interventions in these settings. However, wide-scale variation in care quality persists partly due to the mismatch between CQI interventions and context.

**Methods:** This critical review of implementation frameworks for CQI in Aboriginal and Torres Strait Islander primary health care was conducted in two phases: (1) a review of primary published implementation frameworks used in PHC contexts, and (2) a comparison of key features of these frameworks with quality concepts identified by high-improving Aboriginal and Torres Strait Islander PHC services in remote Australia.

**Results:** We found nine primary implementation frameworks previously used in PHC contexts guiding interventions within and between macro (broader contextual) level; meso (health service) level; and micro (community and inter-personal) level systems. There was commonality between these frameworks and key quality concepts in Aboriginal and Torres Strait Islander PHC. However, none of the frameworks covered all concepts with rare consideration of communities driving health improvement, two-way learning (integrating cultural knowledge into healthcare provision), and caring staff—engendering trusting relationships with community enacted through respect.

**Conclusion:** Respect, as a secret essence, privileges the importance of culture, and is an essential element of CQI implementation frameworks for positive change in Aboriginal and Torres Strait Islander PHC services. It is essential to work with communities to design workforce models that grow a caring stable workforce to ensure improvements in quality of care that are effective for their context.

## Introduction

Primary health care (PHC) services are complex systems, shaped by an interplay of individual, organisational and broader system level factors. Health workers, PHC services and the cultural, social, and political context in which they operate, all interact to deliver health services to people seeking care. For global Indigenous PHC service delivery models, culture is key to all components of PHC delivery: accessibility; community participation; continuous quality improvement (CQI); culturally skilled workforce; flexible approach to care; holistic health care; and self-determination and empowerment (1). Globally, cultural embeddedness and community self-determination distinguish Indigenous PHC services from models of PHC that are not culturally appropriate (1). For Aboriginal and Torres Strait Islander PHC services, culturally embedded services manifests in closer relationships with the people they serve, local knowledge of community, and increased cultural awareness (2). These strengths can unfortunately be countered by workforce issues, especially in rural and remote PHC settings. These issues include limited support and career pathways for Aboriginal and Torres Strait Islander health professionals and high turnover of non-Indigenous health professionals. Limited resources and distance from tertiary health facilities are additional challenges (3).

Continuous Quality Improvement (CQI) has proven to be effective for identifying priority issues, and in health care delivery, for designing, implementing and evaluating improvement interventions in Aboriginal and Torres Strait Islander PHC services. In this setting, there has been wide uptake and sustained use of CQI resulting in improved delivery of care (4–9). However, wide-scale variation in quality of care provision persists, partly due to a mismatch between the implementation of CQI interventions and the health centre context.

**Figure 1:**
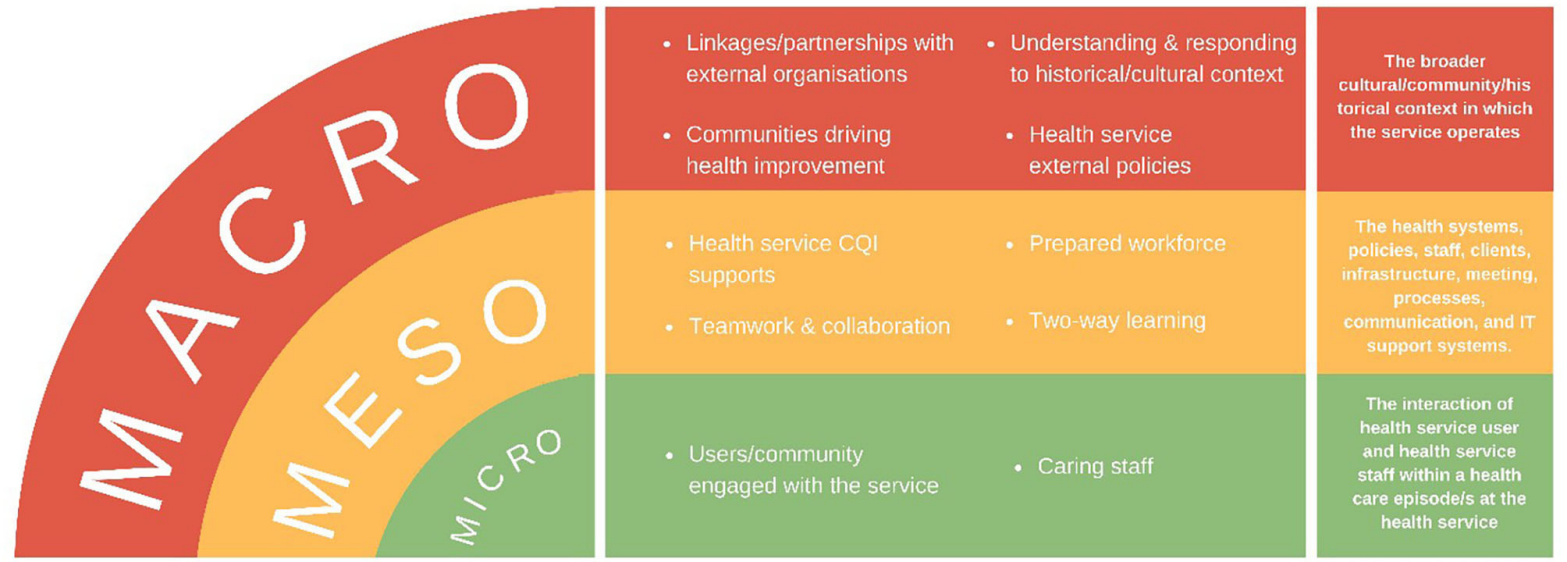
Factors influencing continuous quality improvement (CQI) at high-improving services (10, p. 9).

In previous CQI research “Lessons from the Best,” facilitated with service providers and users of remote PHCs, we documented the “secrets of success,” from consistently high-improving Aboriginal and Torres Strait Islander PHC services of different sizes, governance structures and geographies (10). These high improving services provided appropriate staff orientation and promoted trusting relationships amongst team members and with service users. This approach was key to a stable and engaged PHC workforce, as evidenced in both audit and interview data provided by Indigenous staff who were community members and community members themselves (10). For a number of years prior, these services had utilised a CQI program developed specifically within the Aboriginal and Torres Strait Islander PHC setting (11). The key concepts influencing success of CQI across these services operated at three levels (Figure 1): macro (broader contextual) level; meso (health service) level; and micro (community and inter-personal) level. These high-improving services were responsive enough to modify their activities according to context to optimise quality improvement.

In addition to being adaptive and tailoring improvement strategies to context, successful implementation of interventions has been shown to depend on a number of factors, including well-embedded and consistently applied CQI systems, a stable well-prepared workforce and teamwork (10, 12–14), and skilled CQI facilitation (15, 16). Community involvement in guiding collection and utilisation of CQI data, and in designing strategies to address local priorities, also promotes effective and culturally appropriate improvements in care quality (1, 17, 18).

Implementation frameworks that enable PHC services to enact CQI in partnership with the community could benefit all aspects of health care—clinical and non-clinical. The evidence base for implementing CQI in a participatory way to improve community engagement and cultural safety is not as developed as for clinical CQI, which has well-developed indicators based on best practise guidelines. Further, PHC services may not be appropriately oriented or resourced to work effectively in this way. Thus, there is a need for innovative tools and processes to guide respectful service engagement with community and other system stakeholders in the development, implementation and adaptation of interventions to enhance comprehensive PHC. As a first step, it is necessary to consider implementation frameworks being used in PHC services to assess whether and how they include concepts which have been shown to support high quality Aboriginal and Torres Strait Islander PHC. In this paper, we identify primary published implementation frameworks and examine: What quality concepts, identified in high-improving Aboriginal and Torres Strait Islander PHC services in remote Australia, are evident within published implementation frameworks and what concepts are not included?

## Methods

This paper reports two related phases of work. Phase One is a scoping review of implementation frameworks used in PHC contexts and published in the peer-reviewed literature, and Phase Two the mapping of key features to compare included implementation frameworks with quality concepts identified by high-improving Aboriginal and Torres Strait Islander PHC services.

### Cultural Ways of Working: An Extension to Review Methodology

Cultural ways of working have been woven throughout the methods in this review of the literature. This is not a conventional review; as Indigenous and non-Indigenous practitioners and researchers in Indigenous PHC, we foregrounded Aboriginal worldviews to contextualise the relevance and utility of implementation frameworks to the work of Aboriginal and Torres Strait Islander PHC services. This practical, utilitarian and culturally responsive approach differentiates this review from other reviews, including those that have synthesised the literature around implementation frameworks more generally.

Reflecting a collective approach to work, seven Indigenous and non-Indigenous authors held regular meetings to ensure a shared understanding of the purpose and outputs of the review. Our initial work together was to define “implementation framework” for the purpose of this review. We determined that a framework is a structure, overview, system or plan that, in implementation science, consists of descriptive categories (e.g., concepts or variables), with the relations between them thought to account for what happens or what “is” when interventions are implemented (19). An implementation framework may describe or guide the process of translating research into practise, or it may be used to understand and/or explain what influences implementation outcomes. A framework can also provide a structure to evaluate implementation (20).

### Phase One

In Phase One, a scoping review method was applied to identify and map evidence from primary implementation frameworks used in PHC settings (21). Scoping reviews are used to present a broad overview of existing evidence and can be used to generate hypotheses (22). In order to identify and explore relevant implementation frameworks, three databases—James Cook University's version of Pro Quest Summon; PubMed; and Google Scholar—were searched using the terms: “implementation framework” and “primary health care” by one author (JT). Strengths and limitations of each of these databases were considered (23). A search of the journal *Implementation Science* using the journal search function was conducted also using the same search terms. Black and grey literature stored in the *Australian Indigenous Health InfoNet* (https://healthinfonet.ecu.edu.au/) were then searched using the term “implementation” and experts in the field were invited to contribute literature. These processes enabled a more complete view of all available evidence (24).

### Inclusion/Exclusion Criteria

**Figure 2:**
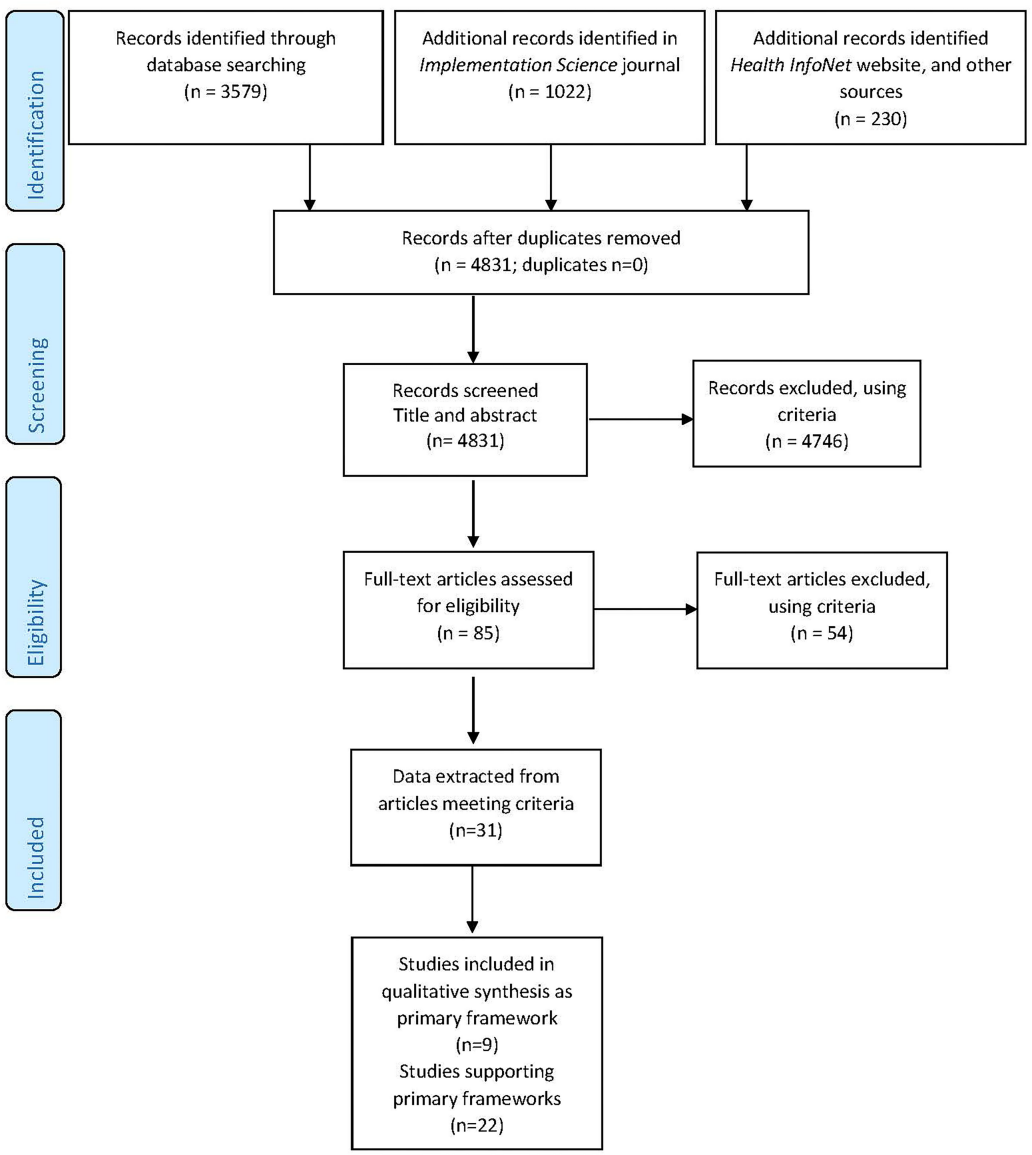
PRISMA-ScR flow diagram (22).

The search strategy followed the PRISMA extension for scoping reviews (PRISMA-ScR) (22) outlined in Figure 2.

Inclusion criteria applied were:

- Included a full description of components of a conceptual framework for implementation of research findings into practise in primary health care.
- Included a full description of components of a conceptual framework for implementation of all types of health interventions/programs/changes into practise in primary health care.

Exclusion criteria applied were:

- Frameworks designed to implement change/intervention for only one type of health condition.
- Written in a language other than English.

No date range was applied to maximise the opportunity to identify primary implementation frameworks.

Our search strategy resulted in 4,831 pieces of literature identified and screened, with no records identified as duplicates. The record title and abstract of 4,831 was read by one author (JT) and if the record met the inclusion and exclusion criteria, details were entered into a shared EndNote^TM^ library. Eighty-five full text records were entered into the Endnote^TM^ library and a full-text assessment conducted for each record against eligibility criteria. A further 54 records were excluded, using criteria. A total of 31 peer-reviewed articles met the inclusion and exclusion criteria, as shown in the PRISMA-ScR Flow Diagram below (Figure 2).

The 31 articles that met the inclusion criteria were entered into a matrix, with articles then grouped around the frameworks they described (JT). The full text for each of the included articles was then re-read and data extracted including: author and year, a full description of the purpose of the framework and a full description of the components of the framework. The nature of the literature was assessed using the Sanson-Fisher et al. typology (25) by a second author (MRM). The methodological quality of included literature was not assessed, consistent with scoping review methods (26).

In recognition of the vast number of implementation frameworks that have been repeatedly used and reported on in health care intervention literature, the 31 identified papers were re-examined (MRM) to determine:

- The primary implementation framework, that is a framework that had been consistently used in the literature reporting on implementation of research findings or interventions; or
- The combined framework, that is a framework that combined two or more primary frameworks;
- The application of a primary or combined implementation framework to a specific context or health condition.

### Phase Two

Consistent with the extended critical review methodology described by Levac et al. (27), four members of the author group (JT, AL, KV, and MRM) compared the primary implementation frameworks with core quality concepts identified from high-improving Aboriginal and Torres Strait Islander PHC services (10). Translational, action-oriented research is both retrospective and normative, according to May and Finch (28). Therefore, we legitimised “looking back” to see what worked in our previous research with high-performing services. After consideration, it was decided that concepts associated with high improvement identified from the “Lessons from the Best” study would be a useful comparator (10).

Key concepts from the “Lessons from the Best” study were re-examined, expanded and contextualised by authors who were also researchers on that study (NNT, VM, JT, and MRM), with critical questioning from others in the author group (QT, AL, and KV). We also reviewed primary interview data from the “Lessons from the Best” study to provide more examples of how concepts were used in the original study. This resulted in a table presenting the factors identified as influencing CQI and a contextualised explanation (Table 1). We respectfully acknowledged that the authors of the implementation frameworks whose work we were reviewing came from different standpoints and PHC contexts.

**Table 1:** Expanded and contextualised concepts from “Lessons from the Best” study.

**LFTB concept**	**LFTB concept expanded and contextualised**
**Macro**	
a) Understanding and responding to historical and cultural context	Health staff need to know and understand the culture and history of the people. Culture is the foundation to everything: relationship to Country, social relationships and individual psychological wellbeing.
	Understanding culture is about understanding the ways things are done, the importance of relationships and obligations, how to exchange ideas, how to share news and how the family and community systems function.
(b) Supportive external health service policies (especially re CQI)	In high improving health services, there is external leadership, training and support for health service staff and workforce policies and tools to facilitate CQI.
(c) Community driving health (care)	The community is in a position to put themselves “in the driver's seat” to actively plan and coordinate health care
(d) Linkages and partnerships with external organisations	High-improving services link with external organisations to strengthen the healthcare they are providing.
**Meso**	
(e) “Two-way” learning for CQI (Indigenous culture and health)	Integrating knowledge about Aboriginal community, family sensitivities, obligations, and traditional ways with effective healthcare and CQI processes—this is “two-way” learning.
f) Prepared and stable workforce for CQI	A prepared workforce includes stable staff, proper orientation, Aboriginal and non-Aboriginal staff, trusting relationships, and supportive leadership.
(g) Teamwork and collaboration: shared focus	A commitment of staff to work together for improved health for the health service users and the community.
(h) CQI systems and supports at health service level	Effective CQI systems are integrated into core business and supported by: information technology and data recording systems; interdisciplinary teams engaged with CQI processes and CQI tools; and regular production of quality of care audit reports to understand and inform system improvements.
**Micro**	
(i) “Going the extra mile” staff caring and commitment	Health staff making every effort to provide the best care, this helps build a trusting and caring relationship between people and the health service.
(j) User/community engaged with the service	Having a good relationship between the community and the health services.

Primary frameworks were divided between authors to “map” in relation to the contextualised concepts from the “Lessons from the Best” study. Each framework was reviewed and mapped twice by separate authors/author groups (some authors were doing a review for the first time) to both ensure quality of the mapping process and that key aspects of the frameworks were included. Where there were discrepancies in mapping outcomes, authors discussed and resolved these to reach consensus.

## Results

### Results Summary

The results reported below reflect the two phases of the critical review: Phase One: search strategies and results, and Phase Two: mapping of included frameworks in relation to “Lessons from the Best” concepts. The authors note that the quality concepts used to map implementation frameworks were developed for different purposes, approaches, and contexts.

We found quality improvement concepts in implementation frameworks applicable within and between macro, meso, and micro-level systems. These concepts reflected many of the quality concepts identified by high-improving Aboriginal and Torres Strait Islander PHC services (10) and could be used in Aboriginal and Torres Strait Islander PHC services to overcome key implementation challenges. However, very little was found about communities driving health improvement, two-way learning, and caring staff—all concepts shown in our previous work to be critical to high improving Aboriginal and Torres Strait Islander PHC systems.

### Phase One

Of the 31 pieces of literature that met the inclusion criteria, we derived nine articles that were the seminal papers originally describing each of the primary implementation frameworks (Table 2). These seminal articles were original research (*n* = 2), reviews (*n* = 3) and discussion papers or commentaries (*n* = 3). One article was a combination of review and original research (33).

**Table 2:** Primary frameworks literature selected for mapping.

**Framework**	**References**	**Summary**	**Descriptive category**
(1) Theoretical Domains Framework	(29)	This is a theory-informed implementation framework designed to identify influences on health professional behaviour as a basis for informing intervention design, often in clinical settings. Changed behaviour is the goal. Following validation, a revised version was published in 2012.	Original research—descriptive
(2) Consolidated Framework for Implementation Research (CFIR)	(30)	Process-orientated conceptual framework designed to guide implementation; CFIR is composed of five major domains: intervention characteristics, inner setting, outer setting, inner setting, characteristics of individuals involved in implementation, implementation process.	Review
(3) Quality Implementation Framework (QIF)	(31)	This framework is a sequence of 4 phases comprising 14 steps for quality implementation. The phases are summarised as: initial considerations regarding the host setting; creating a structure for implementation; ongoing structure once implementation begins; improving future applications.	Review
(4) Knowledge to Action Framework (KTA)	(32)	Steps in implementation process composed of two distinct but related components—knowledge creation (production and synthesis of knowledge) and the action cycle (activities needed for implementation). This is a non-linear model, designed to capture actions and strategies that constitute an effective implementation process.	Review
(5) NASSS Framework: non-adoption, abandonment, scale-up, spread and sustainability	(33)	The NASSS framework comprises seven domains: condition/illness, technology, value proposition, adopter system, organisation(s), wider context, and interaction and mutual adaptation between these domains over time.	Review and original research—descriptive
(6) Promoting Action on Research Implementation in Health Services (PARiHS)	(34)	The PARiHS framework presents the successful implementation of research into practise as a function of the interplay of three elements: the level and nature of the evidence, the context or environment into which the research is placed, and the method or way in which the process is facilitated. All three elements are given equal standing.	Discussion papers or commentaries
(7) i-PARIHS: PARIHS revisited	(35)	A revision of the PARiHS framework, in which the term “innovation” replaces “evidence” and “recipients” is included as a construct. “Facilitation” is positioned as the active ingredient of implementation, assessing and aligning the innovation to be implemented with the intended recipients in their local, organisational, and wider system context.	Discussion papers or commentaries
(8) Normalisation Process Theory (NPT)	(28)	This framework explains the processes involved in health professionals' practises being embedded and becoming normalised. Important concepts include context as a process rather than a place, and collective action rather than individual behaviour the centre of the implementation work.	Discussion papers or commentaries
(9) He Pikinga Waiora Implementation Framework	(36)	The Framework has indigenous self-determination at its core and consists of four elements: cultural-centeredness, community engagement, systems thinking, and integrated knowledge translation. All elements have conceptual fit with Kaupapa Māori aspirations.	Original research—implementation

### Phase Two

Key concepts from the nine primary implementation frameworks were mapped in relation to Larkins et al. (10) concepts in macro, meso and/or microsystems. We mapped these concepts to identify if they were: (i) included in the framework, (ii) not included in the framework, or (iii) partially included in the framework, that is the concept is mentioned in Framework but not completely aligned with LFTB concept (Table 3). We found many quality improvement concepts in the implementation frameworks reflected quality concepts derived from high-improving Aboriginal and Torres Strait Islander PHC services.

**Table 3:** Mapping of primary frameworks.

	**Lessons from the Best study concepts** ***(refer to****Table 1****for further details of each key concept)***									
	***Macro***				***Meso***				***Micro***	
**Primary framework**	**(a)** ** Historical and cultural context**	**(b)** ** External policy and support**	**(c) Community driven**	**(d)** ** Links to external orgs**	**(e)** ** Two-way learning**	**(f) Workforce**	**(g) Teamwork**	**(h)** ** CQI systems**	**(i)** ** Caring staff**	**(j) Community engagement**
(1) Theoretical domains frameworkMichie et al. (29)						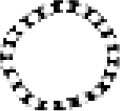	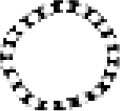			
(2) CFIR: Consolidated framework for implementation researchDamschroder et al. (30)	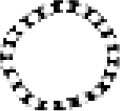					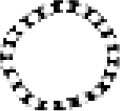				
(3) QIF: Quality implementation frameworkMeyers et al. (31)				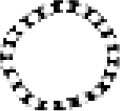						
(4) KTA: Knowledge to action frameworkGraham et al. (32)				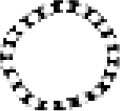			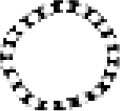			
(5) NASSS Framework: non-adoption, abandonment, scale-up, spread, and sustainabilityGreenhalgh et al. (33)										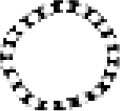
(6) PARiHS: Promoting action on research implementation in health servicesKitson et al. (34)				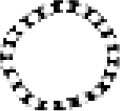		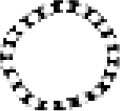				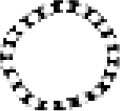
(7) i-PARIHS: PARIHS revisitedHarvey and Kitson (35)						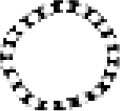				
(8) NPT: Normalisation process theoryMay and Finch (28)		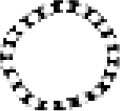					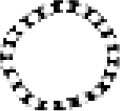			
(9) He Pikinga Waiora implementation frameworkOetzel et al. (36)						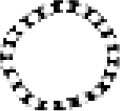				

*

 = concept included in Framework. 
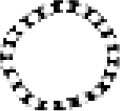
 = concept is mentioned in Framework but not completely aligned with LFTB concept*.

The framework most aligned with key quality improvement concepts identified inductively from high improving services (10), was Oetzel et al.'s (36) *He Pikinga Waiora* Implementation Framework that centralises Indigenous self-determination for chronic disease intervention effectiveness in Aotearoa New Zealand. The Oetzel et al. framework was developed in the context of Maori communities experiencing health inequities in Aotearoa New Zealand. The developers of the framework were aiming for high levels of engagement and service commitment, co-innovation with participants in context (rather than transferring knowledge) and implementation of change at different system levels. Systems thinking and integrated knowledge translation were centralised. Oetzel et al. explain that integrated knowledge translation:

“*supports the communication of new evidence across the system in a manner appropriate for the community and professional setting to improve the quality of services and outcomes for communities*” (29, p. 9).

### Macrosystems

The macrosystems level included important factors outside the health service that impacted implementation of quality improvement. All implementation frameworks identified at least one concept enacted outside of the health service that influenced the successful implementation of quality improvement. Only two frameworks did not mention linkages or partnerships as enabling implementation frameworks. Linkages and/or partnerships with external organisations and professional bodies were identified most commonly (30, 33, 35, 36), with the enabling processes of spanning organisational boundaries (30, 36), sharing visions and information (30), and support of regulatory frameworks, professional and civil society (33) also facilitating implementation of quality improvement. Meyers et al. (31) refer to stakeholder “buy-in” and the need for an innovation to ensure a fit between the setting and the needs of the organisation and/or community.

Understanding and responding to historical and cultural context was described less often in the frameworks reviewed. Graham et al. (32) identified the importance of adapting knowledge or research to the local context. The framework that most explicitly centralised historical and cultural context was Oeztel et al. (36), where a *Kaupapa Māori* approach emphasised Indigenous history, development, and aspirations—cultural respect was key. Critically, this framework clearly described the importance of community being resourced and empowered to actively plan and co-ordinate their health care: *He urunga tangata he urunga pahekeheke, he urunga oneone mau tonu* (the support of others is unreliable, the support of your own is sure) (29, p. 3).

### Mesosystems

Mesosystem, or health service level concepts, were the most commonly described in the implementation frameworks reviewed. CQI systems and supports at a health service level were integral (30–32, 35–37), with a shared focus through teamwork and a prepared and stable workforce described as important for implementing quality improvement. Identifying the roles, processes and responsibilities of team members was important for creating a structure for high quality implementation (31). Most frameworks implied, if not explicitly stated, that a prepared and stable workforce was important for implementing quality improvement. May and Finch (28) outlined a theory to implement and integrate processes that would embed improvement practises. In this theory, collective purposive action was promoted to reshape behaviours or actions of services and individual workers (see section microsystems below). Trust was a key enabler of this action (28).

Few implementation frameworks included the concept of two-way learning. In the Indigenous Australian context, for example, two-way learning integrates knowledge about Aboriginal community, family sensitivities, obligations and traditional ways with effective healthcare and CQI processes (10). The Integrated Knowledge Translation component of the Oetzel et al. (36) framework also included the concept of two-way learning. Turning knowledge into action required co-innovation through the co-design and co-implementation of knowledge and the intervention, led by Maori health workers. This approach to two-way learning is “reflective of Kaupapa Māori and Indigenous self-determination” (29, p. 5).

### Microsystems

Microsystems referred to the relational aspects between health workers and people using the service. Despite the centralising of health service responses in most frameworks, the enabling components of having a good relationship between the community and the health services were rarely described. For example, the Theoretical Domains Framework (38) was individual practitioner focused but not relationally focused. Critical to the results of this review, no implementation framework included explicit reference to the importance of caring staff who are willing to “go the extra mile.” As a key concept described by Larkins et al. (10), a caring approach to service delivery enabled trusting relationships to be established between service users and health professionals—with trust a key concept to lay the foundation for implementing positive change (28, 36).

## Discussion

This review aimed to answer the question: what quality concepts, identified in high-improving Aboriginal and Torres Strait Islander PHC services in remote Australia, are evident within published implementation frameworks and what concepts are not included?

There is clear evidence that concepts described in implementation frameworks have many similarities to those emerging through partnering with highly continuously improving PHC services (10). However, unique concepts identified by high improving Aboriginal and Torres Strait Islander health services also recognised concepts that were referred to only by those working with similar populations or settings (36). Respect is central to all unique quality improvement concepts identified: understanding and responding to historical and cultural context; community driving health improvement; two-way learning; and caring staff are all potential concepts to explore as Aboriginal and Torres Strait Islander PHC services seek to continue to improve the quality of the care they provide for the communities they serve.

In this discussion, the overarching concept of respect as central to implementing CQI is discussed through the perspective of Co-Lead author NNT, an Anmatyerre/Jaru Elder from Central Australia, and key adviser within our author group.

### Respect Is Central to Implementing Quality Improvement: A Positioning of the Results by Nalita Nungarrayi Turner

Respect is the critical underlying issue that drives health service improvement through CQI in Aboriginal and Torres Strait Islander primary health care services in Australia. Nalita Nungarrayi Turner, who has worked with the “Lessons from the Best” team since 2015, explains:

*Without cultural respect there will not be open communication or a positive way of learning. So respect is like a secret essence of culture enabling community people to join with staff to drive their health service. First is the health service user respecting their own and their family's health and looking after themselves. Then there is the health centre staff respecting the cultures of the communities they work with, and finally there is the community/communities respecting the health service and the care they provide. All of these points are interwoven*. -Nalita Nungarrayi Turner (Lead co-author).

Firstly, people respect their health when they have sufficient strengths, individually and at the community level, to be able to think about and act to care for themselves and their family. We know that the need to respect health is interwoven with issues uppermost in family and community relationships, and sometimes health concerns are not the first priority (39). However, the priority for health professionals should always be constant striving to improve and provide appropriate health care. In PHC settings, the mainstream (non-Indigenous), technical language of quality improvement is sometimes inaccessible. However, health professionals talking about and promoting respect, helps the health service user work with their doctor or their Aboriginal and Torres Strait Islander Health Worker or other practitioners for improved health outcomes.

Secondly, it is important to respect the essence of culture and the importance of health service staff behaving in a culturally appropriate way (40, 41). Many health professionals need to shift their ways of knowing; to learn to listen and be open to learning. Staff learning about culture requires that they listen and are guided by Aboriginal and Torres Strait Islander Health Workers, professionals and clients; it requires cultural humility (42). This will help non-Indigenous health professionals to develop a cultural framework for their practise in that community from the ground up. A cultural framework for quality health service delivery includes every aspect of how health services are planned, organised, delivered and evaluated, with the involvement of Aboriginal and Torres Strait Islander people at all stages to implement quality improvement in a health setting (43).

The third aspect, which is the community respecting the health service, is also built on trusting relationships interwoven with understanding and respect (17). Inevitably, there are different ways of knowing and understanding amongst different people in the community and different health service staff. However, while health service delivery may continue if the community do not respect the health service, its ability to deliver an effective service is compromised.

*Understanding the secret essence of respect for culture, in all its aspects, is a steep learning curve for* (non-Indigenous PHC) *staff who come to work in Aboriginal or Torres Strait Islander health services. It doesn't happen overnight—it takes time for people to know what is expected of them when they work in a particular service. The different ways that respect occurs needs to be in balance. There are always ways through which things become unbalanced. Just one of these, focusing on the workforce, is when staff change and a new person comes who is unfamiliar with the ways of doing things. Having a cultural framework, and a cultural guide who can say “People can know what is expected of them when they work in this particular Aboriginal or Torres Strait Islander health service. It is all there, and the rule of the land, and these are the ways we would like you to work,” makes it easier, hence the requirements for a cultural mentor in many Aboriginal and Torres Strait Islander health training frameworks*. -Nalita Nungarrayi Turner (Lead co-author).

These three aspects of respect provide explicit indicators of ways of working and with further work will be able to be measured. The next layer, underneath the framework and how things are shown in practise, is through trusting relationships and moving in safe places and spaces in which to talk with each other and provide health care (17).

### How Do We Use This Wisdom?

This contribution by Nalita Nungarrayi Turner has stressed that it is relational aspects, mediated through respect, that enable service-community partnerships to improve healthcare. While Aboriginal and Torres Strait Islander colleagues know this intuitively, it is not yet clearly operationalised in implementation frameworks to support quality improvement. It is not surprising then, that our exercise of mapping key “Lessons from the Best” concepts against internationally published implementation frameworks resulted in “missing ingredients” centred on service-community relationships. Most implementation frameworks are by necessity generic and are designed for use across a variety of health care settings, often with a clinical-focus, and often by people external to the service setting. Aboriginal and Torres Strait Islander PHC service delivery models, however, are specific to context and exemplify a comprehensive primary health care approach (44, 45). They are characterised by “grass roots” community participation and empowerment and acknowledge PHC as a collective community effort underpinned by cultural perspectives (46).

We know complex workforce, organisation and resourcing factors, alongside the wider community context, combine to influence the success of change interventions (10). Appropriate implementation frameworks, tools and processes are required to aid Aboriginal and Torres Strait Islander health services in CQI endeavours, taking into account the important concepts known to influence the degree to which service quality improves in response to CQI cycles. Quality improvement initiatives might vary greatly depending on who defines quality and thus how it might be measured/assessed (47). A systematic review of interventions in Aboriginal and Torres Strait Islander services (48) found that only one third involved structural changes through, for example, changing policies, systems or organisational and/or community practise. Implementation frameworks used in Aboriginal and Torres Strait Islander health service settings need to be contextually informed and developed in conjunction with relevant populations to ensure concepts that are valued by those populations are included.

Our mapping exercise evidenced that not all key concepts for implementing quality improvement were made explicit in each framework; staff commitment to building a trusting and caring relationship between people and the health service was not included. Workforce was discussed in many of the frameworks included in this review, but the relational aspects of workforce were missing—trust, continuity, two-way learning and mutual respect. This omission might have occurred as the importance of personal relationships and trust are self-evident and thus taken for granted. However, more explicit inclusion of these interpersonal aspects in quality frameworks may assist to reflect the priorities of health service users.

Two-way learning for quality improvement was centralised in the Oetzel et al. (36) framework, but not other the frameworks. Two-way learning in PHC contexts refers to the integration of knowledge about Aboriginal community, family sensitivities, obligations, and traditional ways with effective healthcare and CQI processes. Community involvement, community participation, or community driving health improvements are all sites for two-way learning in quality improvement.

The approach adopted in this review has unearthed something different through centralising the perspectives of the users of the service rather than the providers. With these valuable findings, we now delve more deeply into what might be the underpinnings or secret essence that enables caring staff/workforce and community involvement.

### Where to Next? Future Framework Adaptation for use in Improving Care in Indigenous and Other Contexts

Further exploration of how quality of care can be improved is required. The critical relational areas of trust, respect and continuity and what this means in terms of implementation frameworks need to be determined. It is vital that quality frameworks and implementation frameworks reflect the domains of quality that are considered of highest importance by the relevant communities. Smylie et al. (49) have characterised a disparity between Indigenous knowledge systems (ecologic, holistic, and relational) and Western knowledge systems (reductionist, linear, and objective). Through the foregrounding of Indigenous concepts when implementing quality improvement, current implementation frameworks could become more relevant in Indigenous health care settings.

How can healthcare interventions be made workable and integrated in Aboriginal and Torres Strait Islander contexts (or different contexts where there are different cultures)? Specifically, how can caring aspects of health delivery, two-way knowledge exchange and community driving health be made explicit within an implementation framework to support operationalisation at all system levels? A theoretical model that helps us to understand such processes would be a valuable tool in planning and evaluating the implementation of policy and practise (28). Understanding these processes may enable quality improvement for key health professionals, policy makers, funders (governments) and researchers, who are essential to facilitating applied research (50) for improved health outcomes for all.

### Limitations

The literature search was conducted to identify primary implementation frameworks in PHC. This means database searching was constrained through the narrowing of search terms, the absence of searching truncations of key terms, the use of one author only in the initial data screening process and excluding implementation frameworks from fields other than primary health care. Hence the authors may have missed some primary implementation frameworks. However, the expertise in the author group and contributions from experts in the field confirmed that primary implementation frameworks were identified. Consistent with scoping review methodology, no protocol was made publicly available prior to the review being conducted. It is acknowledged that the implementation frameworks identified in the literature and the “Lessons from the Best” quality concepts used to map these frameworks were developed for different purposes and using entirely different approaches and contexts. However, combining the knowledge gained from these approaches whilst centering Aboriginal voices has generated some novel insights.

## Conclusion

Published implementation frameworks incorporate many of the key concepts identified by high-improving Aboriginal and Torres Strait Islander PHC services at the macro, meso, and micro level. However, very little was found about communities driving health improvement, two-way learning, and caring staff. Respect, as a secret essence, privileges the importance of culture, and is required to enact CQI implementation frameworks for positive change in Aboriginal and Torres Strait Islander PHC services. The relationship between respect and caring staff/workforce, and how this learning can be adapted according to context, requires further research. Specifically, working with communities to design workforce models that grow a caring stable workforce is essential to enacting contextualised quality improvements. Outcomes of such research would extend existing implementation frameworks, such as Oetzel et al. (36), and have application in other Indigenous, non-Indigenous and cross-cultural service delivery contexts.

## Author Contributions

MR-M, NT, JT, AL, KV, SL, and VM conceived the study concept and design. JT sourced the literature. JT and MR-M screened the papers. MR-M drafted the manuscript. NT, JT, AL, KV, QT, SL, KC, ST, RB, and VM contributed to the background, rationale, assessment, and interpretation of frameworks. RB contributed as a critical reviewer of earlier concept drafts. KV, AL, and MR-M mapped frameworks. All authors read and were involved in critically revising the manuscript and approved the final manuscript.

## Conflict of Interest

The authors declare that the research was conducted in the absence of any commercial or financial relationships that could be construed as a potential conflict of interest.
